# Frontal Plane QRS – T Angle Is a Predictor of Ventricular Arrhythmia in Heart Failure With Preserved Ejection Fraction

**DOI:** 10.1111/anec.70062

**Published:** 2025-03-12

**Authors:** Çağrı Zorlu, Barış Açıkel, Sefa Erdi Ömür

**Affiliations:** ^1^ Department of Cardiology Tokat Gaziosmanpasa University Hospital Tokat Turkey; ^2^ Department of Cardiology Tokat State Hospital Toakt Turkey

**Keywords:** echocardiography/methods, electrocardiography/methods, frontal plane QRS, heart failure with preserved ejection fraction, mortality, ventricular arrhythmias, ventricular repolarization parameters

## Abstract

**Introduction:**

Various ventricular repolarization parameters are known to predict ventricular arrhythmias and mortality in various diseases. Although mortality in patients with heart failure with preserved ejection fraction (HFpEF) is similar to that in heart failure with reduced ejection fraction patients, studies on this subject are more limited. Therefore, it is important to evaluate the relationship between ventricular arrhythmias and mortality and ventricular repolarization parameters, especially the frontal plane QRS–T angle, in patients with HFpEF.

**Methods:**

Electrocardiographic, echocardiographic, and laboratory data of 811 patients were evaluated, and the fQRST angle was calculated on ECG. The occurrence of ventricular tachycardia, ventricular fibrillation, or sudden death within a mean of 48 ± 12 months was recorded. Statistical significance was determined as *p* < 0.05.

**Results:**

A total of 811 patients were evaluated, 180 patients in the cardiac event group and 631 patients in the no cardiac event group. NT‐proBNP, La size, La volume index, Tp‐e time, Tp‐e/QTc ratio, and fQRS‐T angle were statistically significantly higher in the cardiac event group. NT‐proBNP level and fQRS‐T angle were found to be independent predictors of mortality in multivariate cox analysis. According to ROC analysis, when QRS‐T angle has a cut‐off value of 58.63, its sensitivity is 81.2, and its specificity is 79.3. Kaplan‐Meier analysis also found that when the fQRS‐T angle was > 58.63, mortality was higher than at narrower angles.

**Conclusions:**

According to our study, the fQRS‐T angle, which can be easily and inexpensively calculated on ECG, predicts long‐term ventricular arrhythmias in patients with HFpEF.

## Graphical abstract

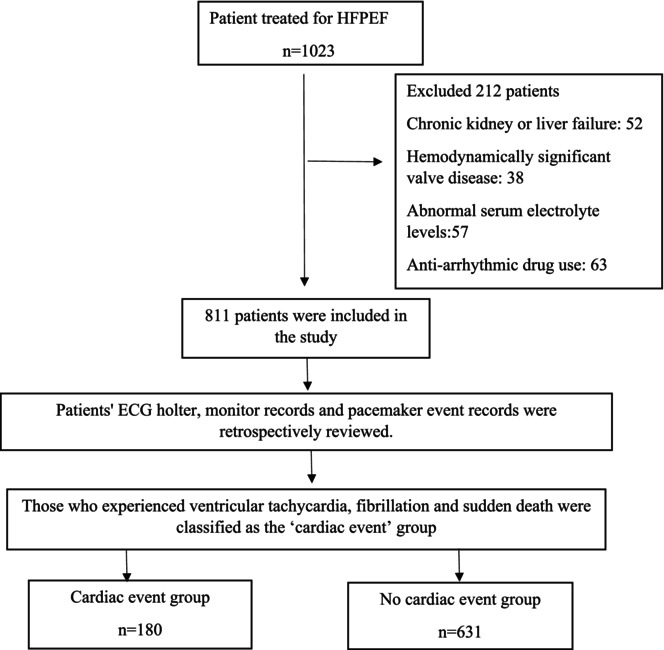

## 1 Introduction

Heart failure (HF) is a disease associated with serious morbidity and mortality, affecting more than 37.7 million people worldwide (Vos et al. 2012). HF with preserved left ventricular ejection fraction (HFpEF), a common form of HF, has attracted increasing attention in recent years due to its high mortality, hospitalization burden, and limitations in treatment.

It has been shown that HFpEF patients, whose number has increased as a result of hypertension, diabetes, obesity, and the aging population, exceed HF with reduced ejection fraction (HFrEF) patients by accounting for approximately 50% of hospital admissions due to HF (Steinberg et al. 2012; Benjamin et al. 2019). Several studies suggest that the annual all‐cause mortality rate of patients with HFpEF varies between 25% and 30% per year, similar to that of patients with HFrEF (Owan et al. 2006; Bursi et al. 2006; Lee et al. 2009). When planning the treatment of patients with HF, it is important to know the prevalence and incidence of arrhythmia types that are likely to be encountered. Sudden death is the most common cause of mortality in patients with HFpEF (Zile et al. 2010). Although sudden death is the most common form of death in patients with HFpEF, less is known about the underlying mechanisms than in patients with HFrEF. When the role of sudden deaths in patients with HFpEF was investigated, it was found that 75% of deaths were related to ventricular arrhythmias (Cho et al. 2018).

Hypertrophy‐related slowing of conduction velocity, delayed repolarization, and increased ventricular fibrosis due to systemic inflammation have been suggested as the mechanisms of ventricular arrhythmias in HFpEF patients (Cho 2022). Abnormalities in ventricular repolarization parameters (QRS, QT, QTc durations, frontal plane QRS‐T angle, Tp‐e duration, Tp‐e/QTc ratio, QTc dispersion), which reflect the distribution of the action potential throughout the myocardium, have been associated with an increased risk of ventricular arrhythmia in the population, with or without structural heart disease (Aro et al. 2012; de Bruyne et al. 1998; Gupta et al. 2008). Abnormal widening of the frontal plane QRS‐T angle (fQRS‐T), defined as the angle between the depolarization and repolarization vectors of the myocardium, has been found to be an indicator of cardiovascular adverse events, arrhythmias, and mortality (Aro et al. 2012; Gotsman et al. 2013). It has been shown that ventricular repolarization parameters change negatively in patients with HF (Son and Boduroglu 2019; Zhang et al. 2021; Morin et al. 2012; He et al. 2023). However, limited data show the relationship between the fQRS‐T angle and other ventricular repolarization parameters with the risk of developing malignant arrhythmia in HFpEF.

In this study, HFpEF patients who had ventricular tachycardia (VT), ventricular fibrillation (VF), or sudden death aimed to determine the relationship between the incidence of mortality and malignant arrhythmias and the fQRS‐T angle and other ventricular repolarization parameters.

## 2 Methods

### 2.1 Patients Population

Patients with HFpEF who were treated for HF symptoms at our center between January 2018 and June 2023 were included in this retrospective study by reviewing their electronic record archives. One thousand six hundred twenty‐one patients with heart failure were evaluated, and 811 patients with HFpEF were included in the study according to the following criteria. The data of the patients over a mean period of 48 ± 12 months were evaluated.

In this patient group:

1. With symptoms and/or signs of heart failure,
2. Left ventricular ejection fraction (LVEF) ≥ 50%,
3. N‐terminal pro‐brain natriuretic peptide (NT‐proBNP) > 125 pg/mL
4. H2FPEF score > 6 in addition to left atrial enlargement and/or increased left atrial volume index (LAVI) was diagnosed with HFpEF by a cardiologist specializing in HF (Reddy et al. 2018).

Patients with VT, VF, and sudden death were divided into 2 groups: the cardiac event group and the no cardiac event group, and ventricular repolarization parameters, especially the fQRS‐T angle, were compared and evaluated.

**FIGURE 1:**
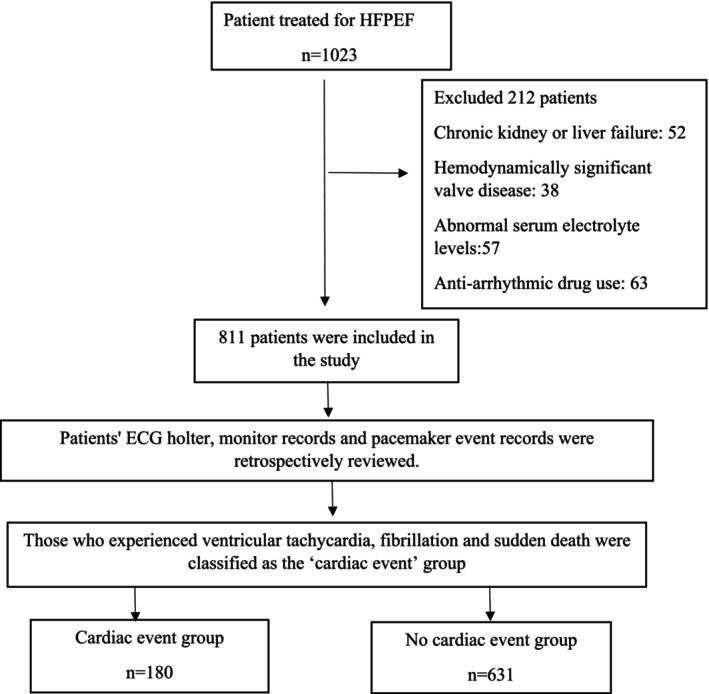
Flow chart of included study participants.

Patients with chronic kidney or liver failure, hemodynamically significant valve disease (moderate to severe aortic stenosis/regurgitation or mitral stenosis/regurgitation), hypertrophic cardiomyopathy, complex congenital HF, isolated right ventricular failures, patients with an ICD or cardiac resynchronization therapy device but whose ejection fraction has improved, heart transplant patients, pericardial disease (cardiac tamponade and constrictive pericarditis) and specific cardiomyopathies (amyloidosis, sarcoidosis, hypertrophic cardiomyopathy, and restrictive cardiomyopathy), anti‐arrhythmic drug use, acute coronary syndrome, cancer patients, sepsis, left or right bundle branch block, ventricular pacing, or non‐specific intraventricular conduction delay on ECG and abnormal serum electrolyte levels were excluded from the study (Figure 1).

Hematological and biochemical values were determined and recorded from venous blood taken after 12 h of fasting.

Chronic renal failure was defined as a glomerular filtration rate below 60 for more than 3 months. Hypertension was defined as systolic blood pressure ≥ 140 mmHg and/or diastolic blood pressure ≥ 90 mmHg measured in the sitting position and/or the use of antihypertensive medication.

Diabetes mellitus was defined as at least two fasting plasma glucose levels ≥ 126 mg/dL or 2‐h plasma glucose levels ≥ 200 mg/dL or glycated hemoglobin (HbA1c) levels ≥ 6.5% or antidiabetic drug therapy used (American Diabetes Association 2021). Low‐density lipoprotein (LDL) > 160 mg/dL or use of statins was considered a diagnosis of hyperlipidemia. Previous coronary artery disease (CAD) was defined as a history of myocardial infarction and/or revascularization, > 50% stenosis in at least one epicardial coronary artery detected angiographically, or evidence of myocardial ischemia on non‐invasive testing; smoking was defined as regular cigarette use.

The study was conducted after receiving local ethics committee approval dated March 14, 2024, and numbered 2024/084, and detailed informed consent was obtained from all participants in accordance with the Declaration of Helsinki.

### 2.2 Echocardiographic Assessment

Echocardiographic measurements were performed on all patients included in the study using a cardiac ultrasound system (Phillips Healthcare). They were performed in standard accepted positions (Nagueh et al. 2016). All reported echocardiographic measurements were averaged over three consecutive cycles. LVEF and left atrial volume were measured in the apical four‐chamber and two‐chamber views according to the modified biplane Simpson's rule and then normalized to body surface area to derive left atrium volume index. Pulsed doppler echocardiography was performed to evaluate the diastolic filling rates of the ventricles in the apical four‐chamber view. Mitral inflow velocity was measured using continuous and pulse wave Doppler from the mitral leaflet tips from the apical 4‐chamber view. Early diastolic mitral peak flow velocity (E), late diastolic mitral peak flow velocity (A), and E/A ratio were measured. The peak early diastolic tissue velocity e′ was measured from the septal and lateral aspects of the mitral annulus. Right ventricular systolic function was assessed by tricuspid annular plane systolic excursion, which was measured by M‐mode echocardiography, obtained by placing the cursor on the lateral tricuspid annulus at the level of the right ventricular free wall in the apical 4‐chamber view in two‐dimensional echocardiography. The peak velocity of tricuspid regurgitation was measured, and pulmonary artery systolic pressure was calculated by summing the maximum gradient of tricuspid regurgitation and the estimated value of right atrial systolic pressure.

All echocardiographic measurements were analyzed by an experienced echocardiologist blinded to clinical data.

### 2.3 Electrocardiographic Assessment

A standard 12‐lead surface ECG (Nihon Kohden, Tokyo, Japan) with a filter range of 0.16–100 Hz, a speed of 25 mm/s, and an amplitude of 10 mm/mV was recorded from the patients in the supine position and evaluated by two independent cardiologists blinded to the characteristics of the patients. All ECGs were transferred to a digital platform, and measurements were made at 400% magnification using Adobe Photoshop software to make more accurate measurements.

**FIGURE 2:**
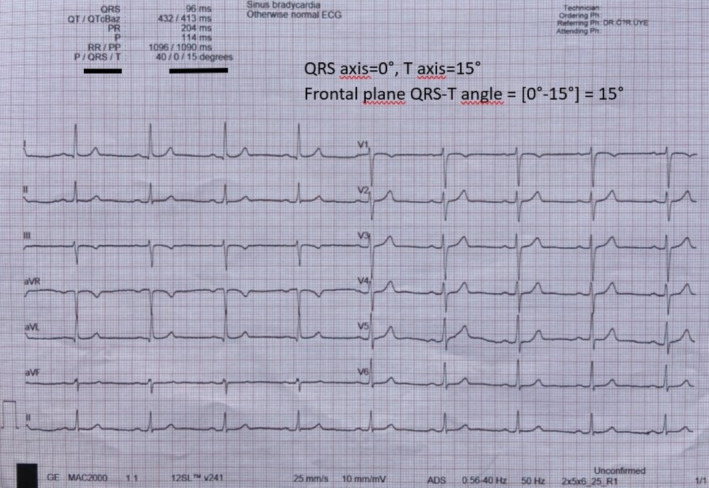
Frontal plane QRS‐T angle measurement from a standard 12‐lead ECG.

P wave duration, PR interval, QRS duration, QT interval, and Tp‐e interval, and QT dispersion were measured manually. QT interval was measured in milliseconds based on the time between the beginning of the QRS complex and the end of the T wave. Corrected QT interval (QTc) was calculated according to Bazett's formula: QTc = QT/√RR. QT dispersion was found by measuring the QT duration in all 12 derivations and calculating the difference between the longest and shortest QT durations. The Tp‐e interval was defined as the time between the top of the T wave and the intersection of the tangent drawn to the downslope of the T wave with the isoelectric line. Tp‐e/QT ratio and Tp‐e/QTc ratio were calculated from the measurements obtained. fQRS‐T angle was calculated as the absolute difference between the QRS axis and the T wave axis obtained from the automatic report section of the ECG device (Figure 2). If the angle exceeded 180°, it was calculated by subtracting it from 360° (Macfarlane 2012).

### 2.4 Evaluation of ECG Holter, Monitor, Pacemaker Records, and Detection of Patients With Sudden Death

The current conditions of the patients were recorded through hospital checks and telephone communication. Monitor records obtained during hospitalization, 72‐h ambulatory continuous ECG monitoring (device brand BTL‐H600) records, and pacemaker histories of the patients were interpreted by board‐certified cardiac electrophysiologists, and malignant arrhythmia (VT, VF) was reported. VT was defined as the occurrence of three or more consecutive premature ventricular complexes at a rate of > 100 beats per minute. VT that is hemodynamically stable and lasts < 30 s is called non‐sustained VT, whereas when there is hemodynamic instability or lasts longer than 30 s, it is called sustained VT. Sudden cardiac death is defined as death thought to be of cardiac cause that occurs within 1 h after the person's cardiac symptoms begin or within 24 h after the person was last seen alive and healthy.

### 2.5 Statistical Analysis

Baseline characteristics of patients with and without malignant arrhythmia were compared. Continuous variables were expressed as mean ± standard deviation or median (25–75 percentile) value, and categorical variables were expressed as percentages and numbers. Categorical variables were compared using chi‐square and Fisher exact tests as indicated. Whether the data were normally distributed was analyzed using the Shapiro–Wilk or Kolmogorov–Smirnov test. Continuous variables were compared with independent samples t‐test or Mann–Whitney *U* test. The Pearson correlation test was used to examine the correlation between variables. Cox analysis models were used to estimate hazard ratios and their 95% confidence intervals for associations between variables and mortality. The receiver‐operating characteristic (ROC) curve was used to determine the sensitivity and specificity of the fQRS‐T and the optimal cut‐off value for predicting mortality. A *p* value of < 0.05 was considered statistically significant. Statistical analysis was performed through the statistical program SPSS (version 22.0; SPSS Inc).

## 3 Results

A total of 811 HFpEF patients were included in this study. Four patients had sudden cardiac death, and 163 patients had non‐sustained VT; 11 patients had sustained VT, and 2 patients had VF. A total of 180 patients were evaluated as the cardiac event group. A comparison of demographics, laboratory, and drug treatments of the patients included in the study is given in Table 1. There was no difference between groups in terms of demographic data and drug treatments. When laboratory data were compared, NT‐proBNP values were statistically higher in the group that developed cardiac events (Table 1). Echocardiographic and electrocardiographic data are given in Table 2. When electrocardiographic and echocardiographic features were compared, LA size, LAVI, QRS duration, P duration, QTc dispersion, Tp‐e, Tp‐e/QTc, and fQRST angle were statistically significantly higher in the cardiac events group and in a total of 17 patients diagnosed with sudden cardiac death, VT, and VF, fQRST angle was 62.51 ± 7.32, *p* < 0.001 (Table 2).

**TABLE 1:** Comparison of demographics, laboratory and drug medications of the groups.

Variable	Cardiac event (*n*: 180)	No cardiac event (*n*: 631)	*p*
*Demographics features*			
Age (years)	68.8 ± 12.0	69.1 ± 11.3	0.579
Female gender (*n*, %)	81 (44.72)	284 (44.96)	0.643
BMI kg/m^2^	30.5 ± 6.4	29.2 ± 3.7	0.535
CAD (*n*, %)	76 (41.38)	265 (41.95)	0.743
Stroke (*n*, %)	10 (5.27)	32 (4.99)	0.351
Diabetes mellitus (*n*, %)	104 (57.77)	359 (56.93)	0.453
Hypertension (*n*, %)	130 (71.94)	435 (68.99)	0.158
Hyperlipidemia (*n*, %)	34 (18.88)	120 (18.95)	0.341
Smoking (*n*, %)	49 (26.94)	158 (24.98)	0.329
*Laboratory findings*			
Glucose (mg/dL)	133.2 ± 18.8	136.3 ± 19.5	0.879
Creatinin (mg/dL)	1.23 ± 0.62	1.21 ± 0.92	0.627
BUN (mg/dL)	53.6 ± 25.4	52.3 ± 23.4	0.490
Sodium (mmol/L)	135.3 ± 6.12	138.1 ± 3.2	0.349
Potassium (mmol/L)	4.82 ± 1.27	4.53 ± 1.67	0.637
Albumin (g/dL)	3.35 ± 1.2	3.43 ± 0.18	0.256
ALT (U/L)	23.45 ± 8.1	22.35 ± 3.4	0.347
AST (U/L)	18.65 ± 7.8	19.63 ± 1.3	0.496
TSH(μIU/mL)	2.57 ± 1.3	1.96 ± 0.97	0.167
T4 (μIU/mL)	1.86 ± 0.6	1.95 ± 0.3	0.472
Hemoglobin (g/dL)	12.19 ± 2.28	13.16 ± 2.24	0.192
WBC count (×10^3^/μL)	11.23 ± 2.34	9.658 ± 2.46	0.083
LDL‐cholesterol (mg/dL)	134.36 ± 45	132.24 ± 12	0.069
HDL‐cholesterol(mg/dL)	35.26 ± 8.6	38.25 ± 3.4	0.093
Triglyceride, (mg/dL)	253.24 ± 42	249.23 ± 23	0.125
Troponin (ng/mL)	38.35 ± 2.9	35.24 ± 1.3	0.158
NT‐proBNP (pg/mL)	3542 ± 458	3020 ± 226	< 0.001
*Drug medication*			
ACEi, ARB (*n*, %)	88 (48.88)	303 (47.97)	0.183
Β blocker (*n*, %)	117 (65)	441 (69.94)	0.139
Furosemid (*n*, %)	176 (97.77)	618 (97.9)	0.539
Spironolactone (*n*, %)	59 (32.77)	202 (31.95)	0.376
SGLT2i (*n*, %)	59 (30)	201 (31.80)	0.283
Anticoagulant (*n*, %)	74 (40.83)	240 (37.98)	0.349
Digoksin (*n*, %)	27 (15)	95 (14.98)	0.861
ASA (*n*, %)	54 (30)	190 (30.05)	0.725

Abbreviations: ACEi, angiotensin‐converting enzyme inhibitor; ALT, Alanin aminotransferase; ARB, angiotensin receptor blockers; ASA, acetylsalicylic acid; AST, Aspartate aminotransferase; BMI, body mass index; BUN, blood urea nitrogen; CAD, coronary artery disease; HDL, high‐ density lipoprotein; LDL, low‐density lipoprotein; NT‐proBNP, N‐terminal brain natriuretic peptide; SGLT2i, sodium‐glucose ko‐transporter‐2 inhibitor; TSH, thyroid‐stimulating hormone; WBC, White blood cell.

**TABLE 2:** Evaluation of echocardiographic and electrocardiographic data.

Variable	Cardiac event (*n*: 180)	No cardiac event (*n*: 631)	*p*
*Electrocardiographic findings*			
QRS (msn)	96.9 ± 26.7	85.9 ± 17.2	0.031
P duration (ms)	88.1 ± 3.07	84.3 ± 5.8	0.028
PR interval (ms)	152.5 ± 33.4	150.8 ± 17.2	0.527
QT (ms)	384.6 ± 61.08	388.1 ± 53.7	0.264
QTc (ms)	445.8 ± 49.96	447.4 ± 45.1	0.347
QTc dispersion (ms)	55.28 ± 5.50	51.23 ± 5.43	0.023
Tp‐e (ms)	87.13 ± 15.9	72.64 ± 16.7	< 0.001
Tp‐e/QTc	0.19 ± 0.03	0.15 ± 0.02	< 0.001
fQRST angle	61.65 ± 8.21	53.52 ± 8.35	< 0.001
	62.51 ± 7.32^a^		< 0.001
*Echocardiographic findings*			
LVEF (%)	52.46 ± 24	53.58 ± 39	0.427
LVDD (mm)	48.12 ± 2.1	47.4 ± 3.1	0.212
LVDS (mm)	35.6 ± 1.8	35.4 ± 0.8	0.374
LA size (mm)	46.9 ± 2.7	43.6 ± 2.1	< 0.001
LAVI (mL/m^2^)	42.2 ± 6.1	35.4 ± 6.3	< 0.001
LVPWT (mm)	11.1 ± 2.3	11.0 ± 0.4	0.536
IVS (mm)	11.2 ± 1.4	11. ± 1.1	0.267
*E*/*e*′	13.8 ± 3.7	13.6 ± 4.1	0.428
PAPs (mmHg)	31.4 ± 7.8	30.8 ± 6.7	0.349
TAPSE (cm)	1.7 ± 0.28	1.7 ± 0.42	0.354

Abbreviations: fQRST angle, frontal plane QRS‐T angle; IVS, interventricular septum; LA, left atrium; LAVI, left atrium volume index; LVDD, left ventricular end‐diastolic diameter; LVEF, Left Ventricular ejection fraction; LVPWT, left ventricular posterior wall thickness; LVSD, left ventricular end‐systolic diameter; PAPs, systolic pulmonary artery pressure; QTc, Corrected QT Interval; TAPSE, tricuspid annular plane systolic excursion; Tp‐e interval, T wave peak to T wave end interval.

^a^ A total of 17 patients diagnosed with sudden cardiac death, ventricular tachycardia, and ventricular fibrillation.

In multivariate cox analysis, NT‐proBNP, LA size, LAVI, QTc dispersion, P duration, Tp‐e, Tp‐e/QTc, and fQRS‐T angle were shown to be independent risk factors for cardiac events (Table 3).

**TABLE 3:** Independent predictors for mortality in patients with HFpEF.

Variable	OR	95% CI	*p*	OR	95% CI	*p*
NT‐proBNP (pg/mL)	1.315	1.118–1.423	< 0.001	1.542	1.406–1.683	< 0.001
QRS (msn)	1.023	0.973–1.042	0.523			
QTc dispersion (ms)	1.059	0.986–1.093	0.004	1.328	1.231–1.637	< 0.001
P duration (ms)	1.541	1.113–1.435	0.003	1.221	1.142–1.650	0.03
Tp‐e (ms)	1.063	1.012–1.096	0.005	1.231	1.096–1.189	< 0.001
Tp‐e/QTc	1.156	1.071–1.347	0.023	1.352	1.196–1.479	0.005
fQRST angle	2.346	1.769–3.459	< 0.001	1.697	1.453–1.987	< 0.001
LA size (mm)	1.406	1.148–1.590	0.032	1.350	1.120–1.550	0.015
LAVI (mL/m^2^)	1.096	1.064–1.125	0.001	1.075	1.013–1.092	0.003

Abbreviations: fQRST angle, frontal plane QRS‐T angle; HFpEF, heart failure preserved ejection fraction; LA, left atrium; LAVI, left atrial volume index; NT‐proBNP, N‐terminal B‐type natriuretic peptide; QTc, Corrected QT Interval; Tp‐e interval, T wave peak to T wave end interval.

**FIGURE 3:**
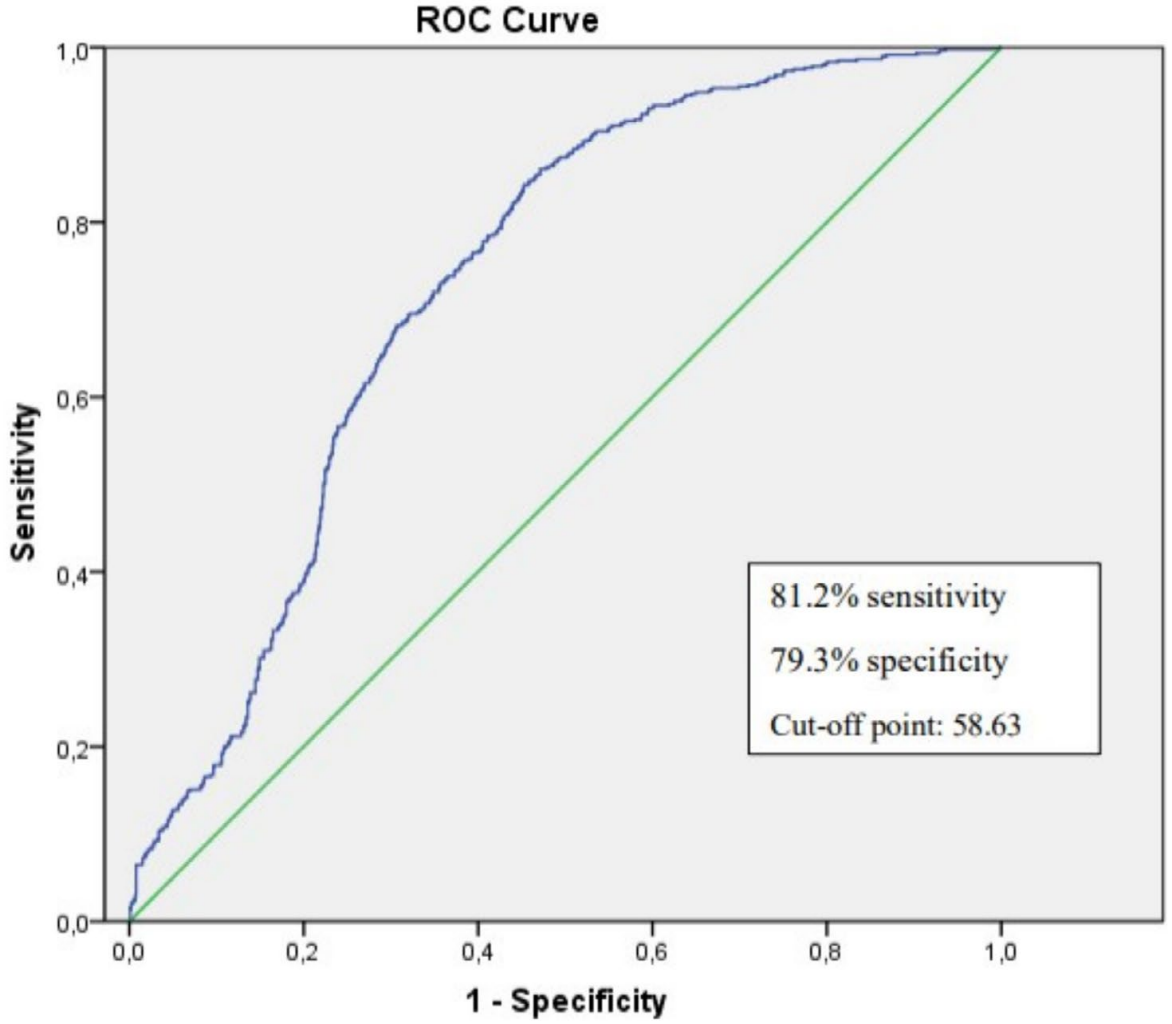
ROC curve analysis of fQRS‐T angle.

**FIGURE 4:**
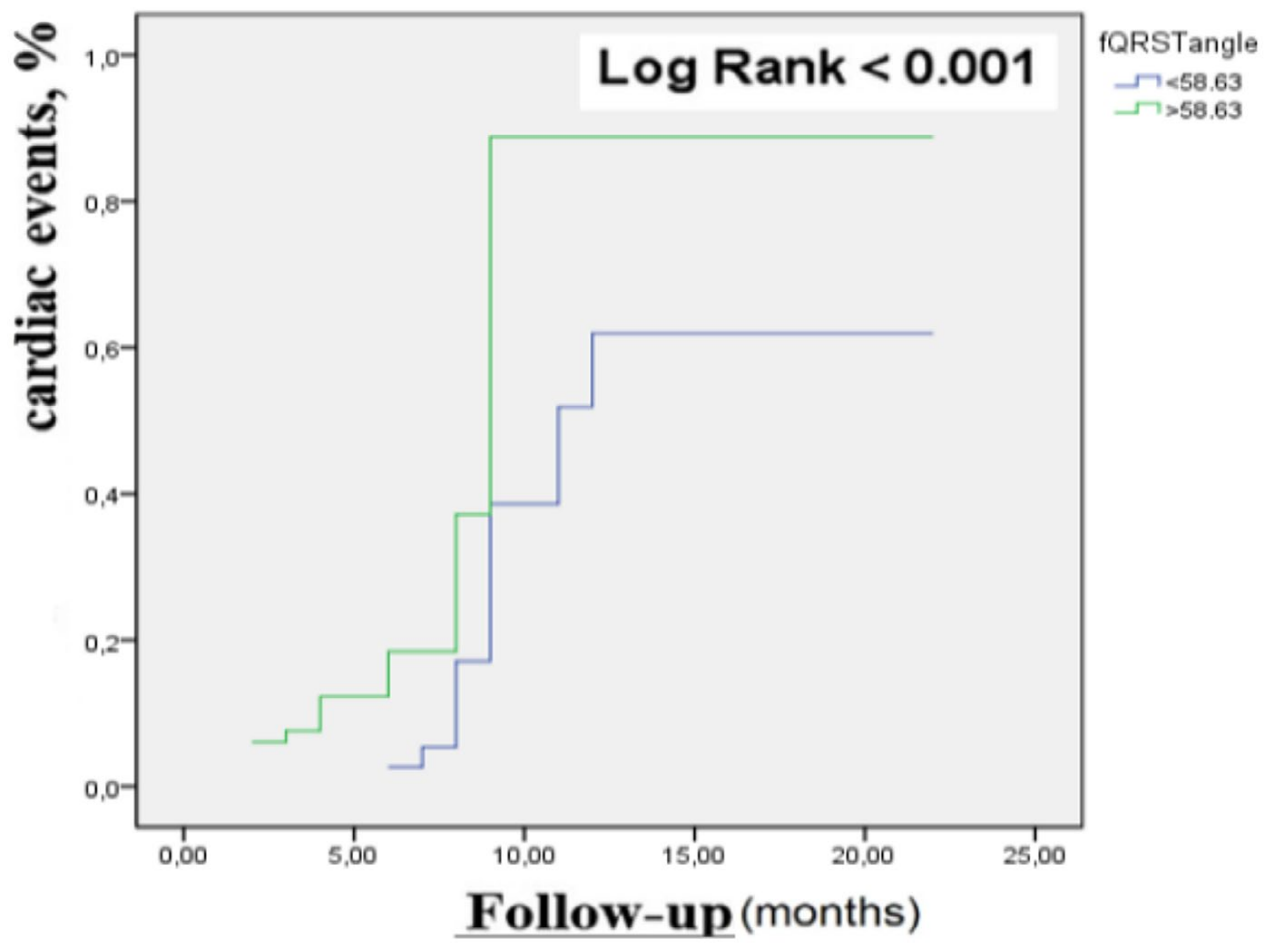
Kaplan Meier analysis of fQRS‐T angle. The cardiac event rate was significantly higher in the wide‐angle group (green line) than in the narrow‐angle group (blue line).

In the ROC analysis, the cut‐off value of the fQRS‐T angle was determined as 58.63 with a sensitivity of 81.2% and a specificity of 79.3% (AUC: 0.731) (Figure 3). According to Kaplan–Meier analysis, major cardiac events were higher in the group with fQRS‐T angle ≥ 58.63 than in the group with fQRS‐T angle < 58.63 (Longrank, *p* < 0.01) (Figure 4).

## 4 Discussion

To our knowledge, although there have been studies evaluating various repolarization parameters, this is the first study to compare QTc, Tp‐e, and Tp‐e/QTc with fQRS‐T angle in patients with HFpEF. We found a more significant relationship between fQRS‐T and VT/VF or sudden death than QTc and Tp‐e/QTC in these patients, and fQRS‐T > 58.63 had an 81.2% sensitivity and 79.3% specificity in predicting ventricular arrhythmia. The fQRS‐T angle was found to be an independent risk factor for predicting long‐term mortality in patients with HFpEF.

It is known that approximately half of patients hospitalized with symptoms and signs of heart failure have normal left ventricular systolic function and diastolic dysfunction and are classified as HFpEF (Benjamin et al. 2019). HFpEF has a similar mortality risk to heart failure with HFrEF (Bhatia et al. 2006). Therefore, studies have investigated the types, incidence, and mortality risks of ventricular arrhythmias in patients with HFpEF (Gutierrez et al. 2020). In our study results, similar to previous studies, NT‐proBNP, QTc dispersion, and Tpe were independent risk factors for mortality in patients with HFpEF.

Previous studies have shown that ventricular repolarization parameters are associated with malignant arrhythmias and have prognostic significance for sudden cardiac death. Several studies have shown that fQRS‐T angle, QTc, QTd, Tp‐e, and Tp‐e/QTc were clarified as signs of increased dispersion of ventricular repolarization on ECG and there are many reports that they predict ventricular arrhythmia and mortality (Akcay 2018; Castro Hevia et al. 2006; Li et al. 2022; Reynard et al. 2019).

A long QTc interval is closely associated with an increased risk of sudden cardiac death in patients without any structural heart disease (Reynard et al. 2019). Long QTc interval and QTc dispersion are potential risk factors for malignant ventricular arrhythmias that affect mortality in different patient groups (Oskouie et al. 2017). It has been reported that the Tp‐e interval is a measure of the transmural distribution of repolarization time and may predict the risk of ventricular arrhythmias (Oskouie et al. 2017). Studies in patients with HFpEF have shown that Tp‐e can be used as a prognostic indicator and is an independent risk factor for mortality (Yan and Martin 2003). In addition, the Tp‐e/QTc ratio has been shown to be a more sensitive index of ventricular arrhythmogenesis. Increased Tp‐e interval and Tp‐e/QTc ratios have been evaluated as electrocardiographic markers of ventricular arrhythmias and cardiovascular mortality (Gupta et al. 2008). Consistent with previous studies, in our study, we found that patients with HFpEF had an increased incidence of adverse cardiac events, including VT, VF, and sudden cardiac death in patients with increased QTc dispersion, Tp‐e, and Tp‐e/QTc ratio.

Increased fQRS‐T angle seems to be related to diastolic dysfunction of the left ventricle. In pathophysiology, reduced expression and function of sodium/potassium‐ATPase have been shown to impair repolarization but, more importantly, also alter intracellular calcium homeostasis, which may worsen cardiac diastolic function (Madias 2011). Increased fQRS‐T angle can be used as the ECG equivalent of abnormal myocardial relaxation. Increased fQRS‐T has been associated with adverse outcomes in patients with HFpEF (Selvaraj et al. 2014). The fQRS‐T angle was examined as a predictive indicator of sudden death in a study of 10,957 middle‐aged patients, and a widened fQRS‐T angle (> 100°) revealed a threefold increased risk of sudden death (Aro et al. 2012). Borleff et al. showed that widening of the fQRS‐T angle predicted ventricular arrhythmias (Borleffs et al. 2009). In our study, the fQRS‐T angle was found to be increased in patients with HFPEF who experienced a cardiac event consisting of VT, VF, and sudden death and predicted mortality in patients with an angle of 58.63 and above.

We found that NT‐proBNP levels were independent predictors of mortality. Previous studies have also shown that there is a linear relationship between high NT‐proBNP levels and hospitalizations and mortality in patients with HFpEF (Verbrugge et al. 2022).

The fQRS‐T angle, which is one of the repolarization abnormalities accepted to predict ventricular arrhythmia, can be easily and inexpensively calculated on ECG, and in our study, we found that the fQRS‐T angle was a stronger independent prognostic indicator of mortality in patients with HFpEF compared to other parameters.

### 4.1 Limitations

The main limitation of the study is that it was retrospective. Another important limitation is the small number of patients included in the study. On the other hand, fibrosis, which is known to be associated with ventricular arrhythmias in patients, could not be elucidated because cardiac MR evaluation was not available at our center.

## Author Contributions

Ç.Z. processed data, performed the statistical analysis, and wrote the paper. S.E.Ö. carried out interpretation of data and was a major contributor in editing the manuscript. B.B., Ç.Z., and S.E.Ö. contributed to the review and revision of the manuscript. All authors read and approved the final manuscript.

## Ethics Statement

The study was conducted after receiving local ethics committee approval dated March 14, 2024, and numbered 2024/084.

## Conflicts of Interest

The authors declare no conflicts of interest.

## Data Availability

The data that support the findings of this study are available from the corresponding author upon reasonable request.
